# Ferricytochrome *c* Directly Oxidizes Aminoacetone to Methylglyoxal, a Catabolite Accumulated in Carbonyl Stress

**DOI:** 10.1371/journal.pone.0057790

**Published:** 2013-03-06

**Authors:** Adriano Sartori, Camila M. Mano, Mariana C. Mantovani, Fábio H. Dyszy, Júlio Massari, Rita Tokikawa, Otaciro R. Nascimento, Iseli L. Nantes, Etelvino J. H. Bechara

**Affiliations:** 1 Departamento de Bioquímica, Universidade de São Paulo, São Paulo, São Paulo, Brazil; 2 Departamento de Bioquímica e Biologia Molecular, Universidade Federal de São Paulo, São Paulo, São Paulo, Brazil; 3 Instituto de Ciências Ambientais, Químicas e Farmacêuticas, Universidade Federal de São Paulo, Diadema, São Paulo, Brazil; 4 Departamento de Física e Informática, Universidade de São Paulo, São Carlos, São Paulo, Brazil; 5 Centro de Ciências Naturais e Humanas, Universidade Federal do ABC, Santo André, São Paulo, Brazil; University of Pecs Medical School, Hungary

## Abstract

Age-related diseases are associated with increased production of reactive oxygen and carbonyl species such as methylglyoxal. Aminoacetone, a putative threonine catabolite, is reportedly known to undergo metal-catalyzed oxidation to methylglyoxal, NH_4_^+^ ion, and H_2_O_2_ coupled with (i) permeabilization of rat liver mitochondria, and (ii) apoptosis of insulin-producing cells. Oxidation of aminoacetone to methylglyoxal is now shown to be accelerated by ferricytochrome *c*, a reaction initiated by one-electron reduction of ferricytochrome *c* by aminoacetone without amino acid modifications. The participation of O_2_ **^•−^** and HO**^•^** radical intermediates is demonstrated by the inhibitory effect of added superoxide dismutase and Electron Paramagnetic Resonance spin-trapping experiments with 5,5′-dimethyl-1-pyrroline-*N*-oxide. We hypothesize that two consecutive one-electron transfers from aminoacetone (E_0_ values = −0.51 and −1.0 V) to ferricytochrome *c* (E_0_ = 0.26 V) may lead to aminoacetone enoyl radical and, subsequently, imine aminoacetone, whose hydrolysis yields methylglyoxal and NH_4_^+^ ion. In the presence of oxygen, aminoacetone enoyl and O_2_ **^•−^** radicals propagate aminoacetone oxidation to methylglyoxal and H_2_O_2_. These data endorse the hypothesis that aminoacetone, putatively accumulated in diabetes, may directly reduce ferricyt *c* yielding methylglyoxal and free radicals, thereby triggering redox imbalance and adverse mitochondrial responses.

## Introduction

Age-related illnesses such as atherosclerosis, diabetes and Alzheimer’s disease have been associated with increased iron and copper release from metal storage proteins and the generation of reactive oxygen and nitrogen species (ROS and RNS) [1]–[23]. These species are known to trigger the peroxidation of lipids, proteins, carbohydrates and DNA ultimately yielding reactive carbonyls such as α-oxoaldehydes, α,β-alkenals, and epoxy-α,β-alkenals, for which conjugation with the nucleophilic amino groups of proteins and nucleobases can lead to a pathogenic condition called “carbonyl stress” [4]. Methylglyoxal (MG) [5], glyoxal [6], acrolein [7], 4-hydroxy-2-nonenal [8], and 3-deoxyglucosone [9] exemplify catabolites that are connected with carbonyl stress. MG produced in cells from triose phosphates, and putatively from aminoacetone (AA) metabolism [10], has been reported to originate ethane cycloadducts with DNA bases and Schiff conjugates with Arg/Lys amino groups of proteins leading to advanced glycation endproducts (AGEs) formation [11]. Methylglyoxal has been implicated in the microvascular alterations that underlie the neuropathy, nephropathy, retinopathy, and atherosclerosis manifested in diabetes [12] and to be associated with the neurological symptoms of Alzheimer’s disease [13].

**Figure 1:**
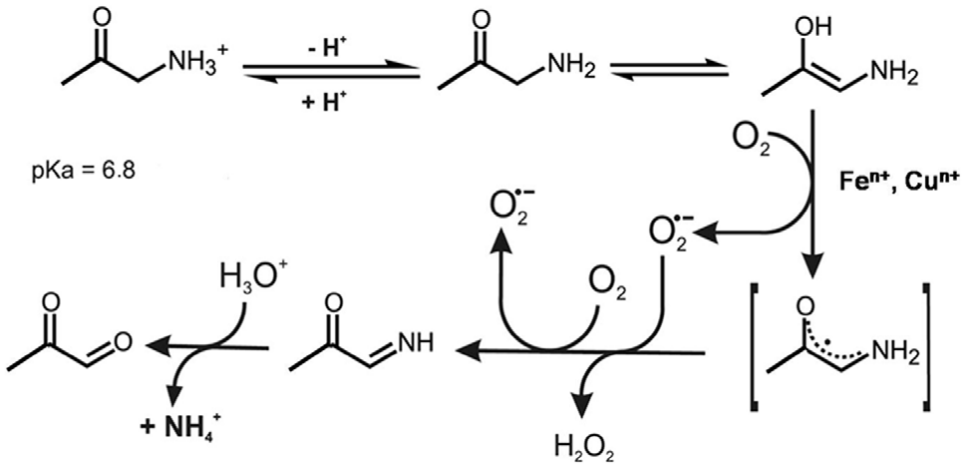
Proposed mechanism of AA oxidation catalyzed by iron and copper ions (Adapted from Dutra et al.[14]).

Conversely, AA is a putative threonine and glycine catabolite produced in the mitochondrial matrix that reportedly undergoes enzymatic and iron/and copper-catalyzed oxidation to MG, H_2_O_2_, and NH_4_^+^ ion [14] (Figure 1). AA enzymatic oxidation is accomplished by a non-specific semicarbazide-sensitive amine oxidase (SSAO) [15] found at high activity levels in the plasma of type I and II diabetics [16]. Dutra et al. [14], [17]–[18] reported that AA *in vitro* promotes oxygen-dependent copper and iron release from horse spleen ferritin and human plasma ceruloplasmin concomitantly with protein modification and function losses. AA is also associated with a variety of injuries in biomolecules and with mitochondrial and cell dysfunction and apoptosis [14], [19]–[20]. Among other α-aminoketones of biological interest that can produce reactive α-oxoaldehydes and ROS by aerobic oxidation, we mention 5-aminolevulinic acid (ALA) [21], a heme precursor accumulated in porphyric disorders, and 1,4-diaminobutanone (DAB), a wide spectrum microbicide [22], [23].

With regard to mitochondrial protein and DNA damage in aging, diabetes and neurodegenerative diseases, the multifaceted, beneficial and harmful roles of cytochrome *c* in electron transport, peroxidatic reactions and apoptosis are documented in detail [24]. Ferricytochrome *c* (ferricyt *c*) can react with lipid-derived peroxides and aldehydes yielding free radical intermediates and triplet carbonyl products, which can promote oxidative damage in a number of biomolecules, including the heme protein itself [25]–[26]. On the other hand, the ferricyt *c* ability to be promptly reduced by the O_2_ **^•−^** radical may contribute to prevent the initiation of deleterious radical chains because the competitive formation of H_2_O_2_ by superoxide dismutation can be lessened [27].

Considering that (i) mitochondrial dysfunction appears to be implicated in the pathophysiology of diabetes [28], (ii) pro-oxidant AA is putatively biosynthesized in the mitochondrial matrix [29], (iii) aminoacetone degradation has been proposed as a contributing source of plasma MG under normal conditions [30], and (iv) the reduction potential of ferricyt *c* [31] and of AA measured here are thermodynamically favorable to one-electron oxidation of AA yielding a resonant AA**^•^** enoyl radical, which is expected to initiate a radical oxidation chain by molecular oxygen, we embarked on an investigation of the reaction mechanism of AA aerobic oxidation initiated by ferricyt *c* and the structural susceptibility of the hemeprotein to the reaction radical intermediates and the final product, MG.

## Materials and Methods

### Reagents

Reagents of the highest available purity were purchased from Sigma-Aldrich (St. Louis, MO), and HPLC quality solvents were acquired from Merck (Darmstadt, GE). AA.HCl was prepared according to Hepworth [32] and recrystallized from ethanol:ether (8∶2). Light yellow AA crystals [34% yield; δ (ppm), in D_2_O: 2.08 (3H, s), 3.88 (2H, s)] were weighed, sealed in Eppendorf vials, placed in a nitrogen glove box and stored at −20°C. Stock solutions of AA were prepared in nitrogen-purged Milli-Q purified water immediately before use. Stock solutions of horse heart ferricyt *c* type III (1.0 mM) were obtained by dissolving the heme protein in Milli-Q purified water. All of the experiments were performed in 50 mM Chelex-treated phosphate buffer, pH 7.4, prepared with Milli Q water.

### Oxygen Uptake

Oxygen uptake was monitored in a Hansatech Oxygraph equipped with a Clark-type electrode. Oxygen consumption by AA (1.0–5.0 mM) was monitored for 30 min at 37°C in the absence and presence of ferricyt *c* (50 µM). Involvement of ROS in the reaction mechanism was verified upon the pre-addition of the antioxidant enzymes catalase (5 µM) and copper-zinc superoxide dismutase (CuZnSOD) (50 U/mL) in the reaction mixture.

### EPR Spin-trapping

EPR spin-trapping studies of the AA/ferricyt *c*-containing reaction mixtures were performed with 5,5′-dimethyl-1-pyrroline *N*-oxide (DMPO) (25–400 mM) and alpha-phenyl-*N*-*tert*-butyl nitrone (PBN) (50 mM) in the presence or absence of dimethyl sulfoxide (DMSO) or ethanol (30%) at 25°C, using a Bruker EMX spectrometer. The EPR spectra were traced 4 min after the addition of 15 mM AA. Catalase (15 µM), CuZnSOD (150 U/mL), desferoxamine (100 µM) and diethylene triamine pentaacetic acid (DTPA) (100 µM) were added to the reaction mixture in order to verify contribution of adventitious iron to the generation of ROS by the complete system. The operating conditions are indicated in the figure legends. EPR spectra were analyzed using the EasySpin program [33], which is frequently employed for the simulation of liquid- and solid-state EPR, including both Gaussian and Lorentzian line shapes.

### UV-Vis Spectrophotometry

Absorption spectra of the samples were recorded with a Varian Cary 50 Bio spectrophotometer at 37°C. Ferricyt *c* (5.0–100 µM) spectral changes during reactions with AA (0.5–30 mM), in the presence or absence of CuZnSOD (50 U/mL), were analyzed by monitoring the bathochromic shift of the Soret band (409 nm) and the increase of the 550 nm absorption band.

### Kinetic Measurements

The initial rates of ferricyt *c* (50 µM) conversion to its ferrous form upon addition of AA at increasing concentrations (0.50–30 mM) (*k*_obs_) were spectrometrically followed at 550 nm, from which the apparent second order rate constant (*k*_2_) was evaluated. All experiments (triplicates) were performed in normally aerated Chelex-treated 50 mM phosphate buffer, pH 7.4, at 37°C.

### Cyclic Voltammetry

Electrochemical studies of AA (5.0 mM) were conducted under strictly anaerobic conditions in DMSO purged with pure nitrogen. The cyclic voltammograms were traced at 25°C, at a scan rate of 100 mV/s. A platinum working electrode was employed in all of the experiments, with a platinum foil as a counter electrode. Potentials were referred to an Ag/AgCl (1.0 M KCl) electrode (+0.503 V) versus a Standard Hydrogen Electrode (SHE). Tetrabutylammonium perchlorate (0.10 mol/L) was used as a background electrolyte.

### Raman Spectroscopy

Ferricytocrome *c* (50 µM) was incubated for 2 h in the presence and absence of AA (5.0 mM), or H_2_O_2_ 200 µM, or MG (200 µM). Before recording the Raman resonance spectra, ferricyt *c* was filtered (Amicon Ultra 10K device), and the pellet was resuspended in 50 mM Chelex-treated phosphate buffer, pH 7.4. Resonance Raman spectra were recorded at 413.1 nm (Kr^+^ ion laser, Coherent INNOVA 90) using a Jobin-Yvon T64000 triple spectrometer, with a liquid nitrogen-cooled CCD detector. The spectral resolution was 4 cm^−1^, and the laser power was maintained at 25 mW. A homemade spinning cell was used to prevent local heating.

### Product Analysis

Methylglyoxal in the spent reaction mixture was derivatized with 1,2-diaminobenzene to form a stable product, 2-methylquinoxaline, and analyzed by HPLC/diode array detection, using a procedure adapted from Deng and Yu [34], as follows. The reaction mixture contained 5.0 mM AA and 50 µM ferricyt *c* or 30 µM FeSO_4_.EDTA prepared immediately before use, dissolved in air-equilibrated 50 mM phosphate buffer, pH 7.4, at 37°C, and prepared in an Eppendorf flask with minimal headspace, where dissolved oxygen is expected to be roughly 200 µM. After 2 h of reaction, 1.0 M HClO_4_ and 1.0 mM 1,2-diaminobenzene were added to stop the reaction and stabilize the product. In all experiments, MG was determined in parallel to control runs (in the absence of AA or fericyt c) using 1,2-diaminobenzene or 2-methylquinoxaline (internal standard) heated at 60°C for 3 h, followed by HPLC/diodo array analysis with detection at 315 nm. No change of 2-methylquinoxaline (B.P. 245–247°C) concentration was observed in the control HPLC traces, thereby discarding the possibility of product degradation by heating [14].

### Tryptophan Fluorimetry

Ferricyt *c* (50 µM) was treated with (1.0–5.0 mM) AA at 37°C for 4 h, and an aliquot of 300 µL of the reaction mixture was removed and filtered (Amicon Ultra 10K device). Protein remaining on the filter was resuspended in 50 mM Chelex-treated phosphate buffer, pH 7.4. This step was necessary to eliminate the interference of AA oxidation products in the fluorescence measurements. The fluorescence intensities of the tryptophan residue of cytocrome *c* (λ_em_ 340 nm, λ_exc_ 280 nm) were measured in a microplate reader (Molecular Devices, model Spectramax M2e).

### CD Analysis

Ferricyt *c* (60 µM) was treated with AA (1.0–5.0 mM) at 37°C for 12 h, diluted 5 times in Milli-Q purified water and then analyzed. The CD spectra were collected in the range 190–630 nm (Far-UV, Near-UV, and Soret regions) in a Jasco J-720 spectropolarimeter, at room temperature. Quartz cells with 0.10- and 0.50-cm light paths were used for measurements in the far-UV and in the near-UV/Soret regions, respectively. All spectra were corrected by subtracting the corresponding backgrounds. The spectra were acquired with 10 nm/min resolution, applying an average of 4 scans per spectrum. The CD spectrum of ferricyt *c* (60 µM) treated with ascorbate (5.0 µM) was traced in parallel to depict the total reduction of the protein to its ferro form.

### Low-temperature EPR Spectrometry

Ferricyt *c* (300 µM) in the presence and absence of AA (1.0–5.0 mM) was incubated in the buffer at 37°C for 12 h before recording the EPR spectra. EPR continuous wave spectra were recorded in a standard rectangular cavity-equipped X-band spectrometer (Bruker Elexsys line E-580). The temperature of ∼11 K was maintained by liquid helium (Helitran Oxford Systems). The samples were placed in a quartz tube and frozen in liquid nitrogen prior to introduction into the microwave cavity for spectral recording. The experimental parameters were set as follows: microwave frequency, 9.5 GHz; microwave power, 5.05 mW; magnetic field scan range, 35–425 mT; and modulation amplitude, 1 mT.

### Magnetic Circular Dichroism (MCD) Spectrometry

Measurements of ferricyt *c* (40 µM) in the absence or presence of AA (1.0–5.0 mM) were conducted in a Jasco J-720 spectropolarimeter. The magnetic field was 870 mT, and the optical path was 5 mm. The spectra were recorded at room temperature, pH 7.4, after 12 h of ferricyt *c* treatment with AA (1.0–5.0 mM). MCD traces with AA (1.0 mM) were also obtained after 1 and 2 h of treatment.

### Preparation of Liposomes

Cellular and mitochondrial mimetic membranes were prepared from stock chloroform solutions of soybean phosphatidylcholine (PC) (2.0 mM) alone, and from a mix of PC (0.85 mM), dipalmitoyl phosphatidylethanolamine (PE) (0.65 mM), and cardiolipin (CP) (0.50 mM), respectivelyh The solvent was evaporated by flushing with N_2_ to allow for the formation of a homogeneous dry film. Lipid films were stored in the dark in a vacuum to eliminate traces of chloroform. Multilamellar vesicles were prepared by mixing 50 mM phosphate buffer, pH 7.4, with the lipid film, followed by an ultrasonic water bath for 5 min at 35°C. Next, unilamellar liposomes were prepared at room temperature by extrusion from the previous multilamellar suspension in a Mini-Extruder (Avanti Polar Lipids, Inc., Alabaster, AL, USA) through 0.1-µm mesh polycarbonate membranes.

### Liposome Peroxidation Measurements

Liposomes were incubated with ferricyt *c* (50 µM) in the presence or absence of AA (1.0–5.0 mM), 1.0 mL final volume, at 37°C, for 2 h. Malondialdehyde (MDA) production was analyzed in a Waters HPLC equipped with a 515 HPLC pump, 474 scanning fluorescence, and 515 photodiode array detectors. MDA separation was performed in a reverse-phase column C-18 (150×4.60 mm, Phenomenex®) with a mixture of 20 mM phosphate buffer, pH 7.0, and with methanol (70∶30 v/v) as the mobile phase. The isocratic flow rate was maintained at 0.60 mL/min, and the analyte absorbance was monitored at 532 nm in 30-min runs. The results are expressed as a fold change relative to control groups.

### Statistical Analysis

All the experiments were performed at least in triplicate. The results were analyzed by One-Way ANOVA, using the Tukey’s Significant Test (Origin version 8.0). A probability of p<0.05 was used as the criterion for statistical significance.

## Results

### Ferricyt *c* Reaction with AA is Accompanied by Oxygen Consumption and Free Radical Generation

Similar to other α-aminoketones such as ALA, a heme precursor, and DAB, a *Trypanosome cruzi* toxin, AA undergoes phosphate-catalyzed enolization followed by metal-catalyzed oxidation, which is propagated by O_2_ **^•−^** and enoyl AA radicals (Figure 1), to ultimately produce MG, H_2_O_2_, and NH_4_^+^ ion [21]–[23] (Eq. 1).
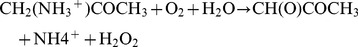
(1)


**Figure 2:**
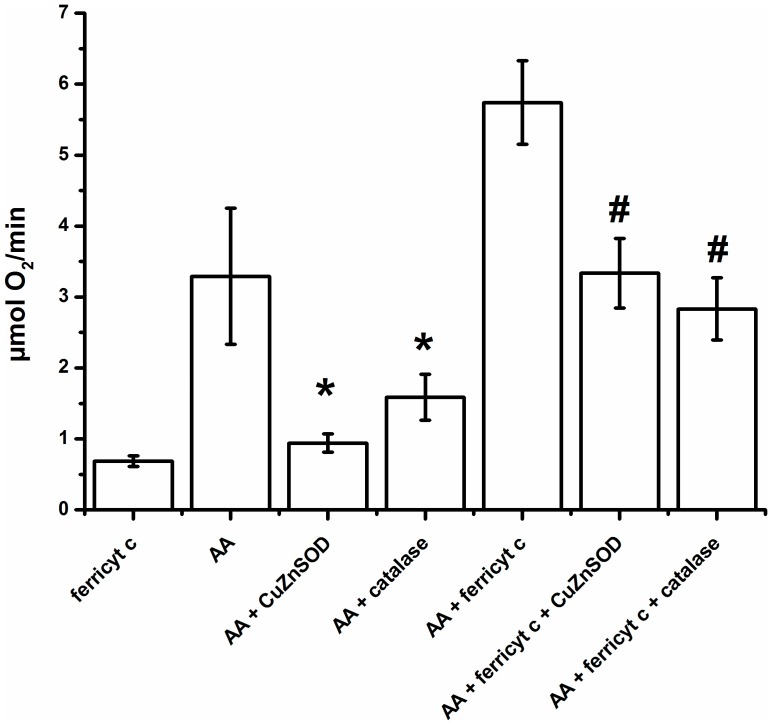
Oxygen uptake by AA in the presence of ferricyt *c*. Experimental conditions: (50 µM) ferricyt *c* in the presence or absence of (5.0 mM) AA in 50 mM phosphate buffer, pH 7.4, at 37°C for 30 min. Experiments were performed in the absence or presence of catalase (5.0 µM) or CuZnSOD (50 U/mL). Data are representative of five independent runs. *p<0.05 relative to the system containing only AA and #p<0.05 relative to the AA/ferricyt *c* system.

Figure 2 shows that AA (5.0 mM) consumes the dissolved O_2_ at a significantly augmented initial rate upon the addition of ferricyt *c* (10 µM): 5.6±0.7 µM O_2_/min (n = 5) *vs.* 3.0±0.9 µM O_2_/min (n = 5). The addition of catalase (5 µM) or CuZnSOD (50 U/mL) to the complete system inhibit oxygen consumption by 50% (2.8±0.4 µM O_2_/min, n = 5) and 40% (3.4±0.6 µM O_2_/min, n = 5), respectively, indicating the involvement of H_2_O_2_ and O_2_ **^•−^** in the mechanism of AA oxidation induced by ferricyt *c*. For comparison, the reaction of ferricyt *c* (50 µM) reduction by AA (1.0 mM), was found to be roughly 10-fold faster and 2-fold slower than those observed with two other α-aminoketones, - ALA and DAB-, respectively, both at 1.0 mM concentration in 50 mM phosphate buffer, pH 7.4 at 36°C (data not shown). At this pH, the enol form of ALA is a carboxylate anion [CH(NH_2_) = C(OH)CH_2_CH_2_COO**^−^**; pKa –COOH ∼ 4.5] [35], DAB is an alkylammonium cation [CH(NH_2_) = C(OH)CH_2_CH_2_NH_3_^+^; pKa –NH_3_^+^ ∼ 9.5] [22], and AA is present in the neutral form [CH(NH_2_) = C(OH)CH_3_]. How the ionic character affects the aminoketone reduction potential, its affinity for cyt c and reaction rates awaits further studies.

**Figure 3:**
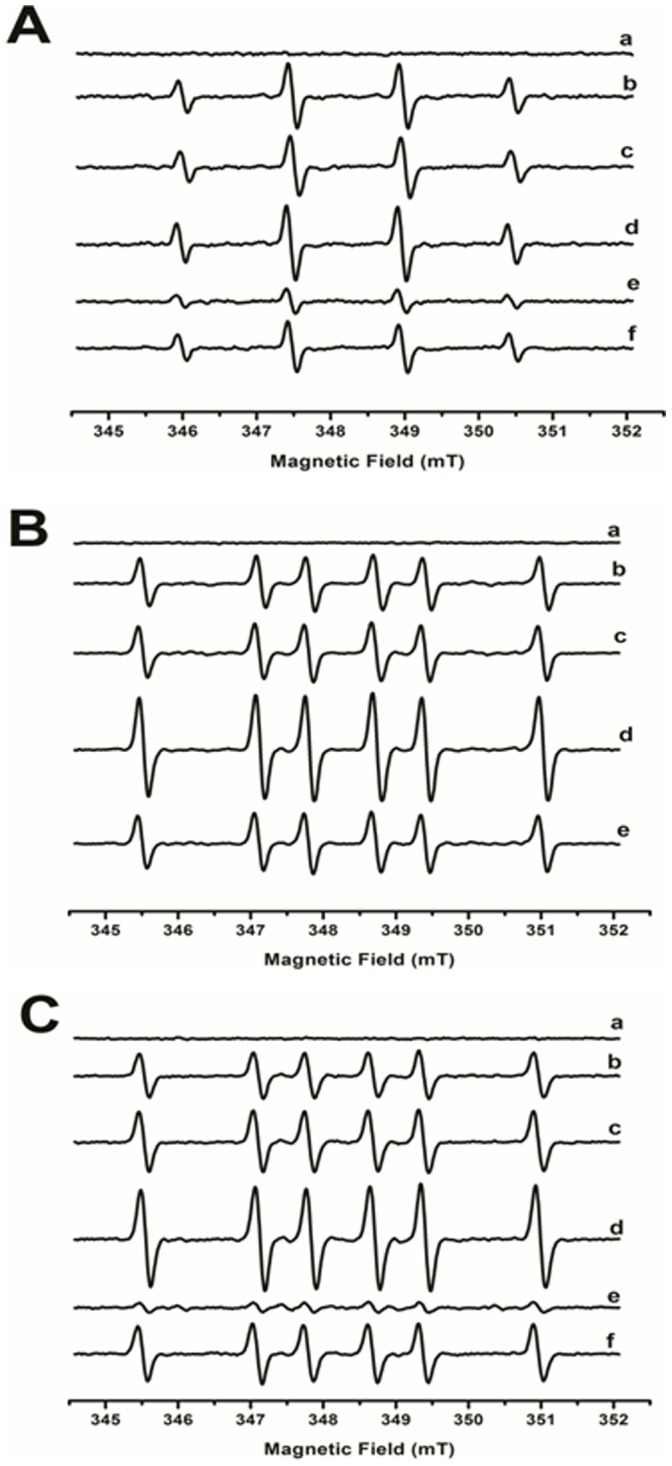
EPR spin-trapping studies of the ferricyt *c*/AA system under aerobic conditions. EPR spectra of DMPO-radical adducts were obtained after a 4-min incubation of 15 mM AA at 25°C in 50 mM phosphate buffer (pH 7.4) with (25 mM) DMPO: (A) DMPO experiments, (B) DMPO in the presence of DMSO 30% v/v, (C) DMPO in the presence of ethanol 30% v/v. For all of the figures: (a) control with ferricyt *c* (150 µM); (b) AA (15mM); (c) AA (15 mM)+desferoxamine (100 µM); (d) ferricyt *c* (150 µM)+AA (15 mM); (e) system d+CuZnSOD (50 U/mL); (f) system d+catalase (15 µM) for Fig. 2A and 2C only. Instrumental conditions: microwave power, 20.2 mW; modulation amplitude, 1.0; time constant, 1.63 s; scan rate 0.1 G/s; and receiver gain, 1.12×106.

That O_2_ **^•−^** and HO**^•^** radicals are formed during the aerobic oxidation of AA both in absence and the presence of ferricyt *c* was demonstrated by EPR spin-trapping experiments with DMPO (Figure 3A). Accordingly, upon incubation of 15 mM AA system with 25 mM DMPO, the characteristic 4-line signal (1∶2:2∶1) of the DMPO-**^•^**OH radical adduct was observed (a_N_ = a_H_ = 1.49 mT) (Figure 3A, trace b) [36]. The DMPO-**^•^**OH adduct probably results from the spontaneous, rapid decay of the DMPO-superoxide adduct (DMPO-**^•^**OOH), whose *t_1/2_* = 27 s and 91 s at pH 9 and 5, respectively [37]. This signal was significantly intensified in the presence of 150 µM ferricyt c (Figure 3A, trace d) that has been shown to accelerate the oxygen-consuming AA reaction (Figure 2). To preclude participation of contaminant iron in the generation of hydroxyl radicals by a Haber-Weiss reaction, parallel experiments were run in the presence of 100 µM desferoxamine or DTPA, which were shown to not decrease the EPR amplitude signal (Figure 3A, trace c). Expectedly, the addition of CuZnSOD (150 U/mL) strongly abated the DMPO-**^•^**OH signal (Figure 3A, trace e). Conversely, the inhibitory effect of catalase (Figure 3A, trace f), although weaker than with CuZnSOD, implies concomitant HO**^•^** radical formation from H_2_O_2_. Possibly, the resonant enoyl AA**^•^** radical intermediate behaves like a semiquinone by donating an electron to H_2_O_2_ yielding HO**^•^** radical, as recently demonstrated by Shang *et al.* when studying the redox cycling of 1,4-naphtoquinone [38]. Accordingly, DMSO addition to the reaction mixture resulted in a signal attributable to the DMPO-**^•^**CH_3_ adduct (a_H_ = 2.24 mT; a_N_ = 1.59 mT) [39], which is reportedly originated by hydroxyl radical-promoted methyl radical release from DMSO (Figure 3B). Additional experiments were conducted in the presence of CuZnSOD and catalase to demonstrate primary formation of superoxide radicals by the reaction of AA with ferricyt *c*. Expectedly, CuZnSOD addition was highly efficient in decreasing the signal of the DMSO-derived methyl radical (Figure 3B, trace e), whereas 15 µM catalase had little effect on the EPR signal amplitude obtained with 150 µM ferricyt *c* and 15 mM AA (data not shown). Because DMSO is known to lessen the catalase activity [40], ESR spin-trapping experiments were performed using DMPO/ethanol to demonstrate that H_2_O_2_ is the main source of HO**^•^** radical in the AA/ferricyt *c* system (Figure 3C). Ethanol is known to be harmless to catalase and can be oxidized by HO**^•^** radical to an α-hydroxyethyl radical yielding the stable adduct DMPO-**^•^**CHOH-CH_3_ (a_H_ = 2.28 mT; a_N_ = 1.58 mT) [41]. The spin signals decreased upon CuZnSOD or catalase addition (Figure 3C, traces d and e, respectively), which corroborates the generation of O_2_ **^•−^** and HO**^•^** radicals by the mechanisms described above.

Further EPR spin-trapping experiments with PBN (α-phenyl-*N*-*tert*-butyl nitrone) were conducted using AA/DMSO to confirm O_2_ **^•−^** and HO**^•^** radical involvement in the AA/cyt *c* reaction. A 6-line EPR signal assignable to the PBN-**^•^**CH_3_ adduct (a_H_ = 0.36 mT; a_N_ = 1.65 mT) was recorded, as previously described by Burkitti and Mason [42] (data not shown). As expected from the DMPO-containing experiments, the PBN-**^•^**CH_3_ EPR signal grew less upon addition of CuZnSOD.

**Figure 4:**
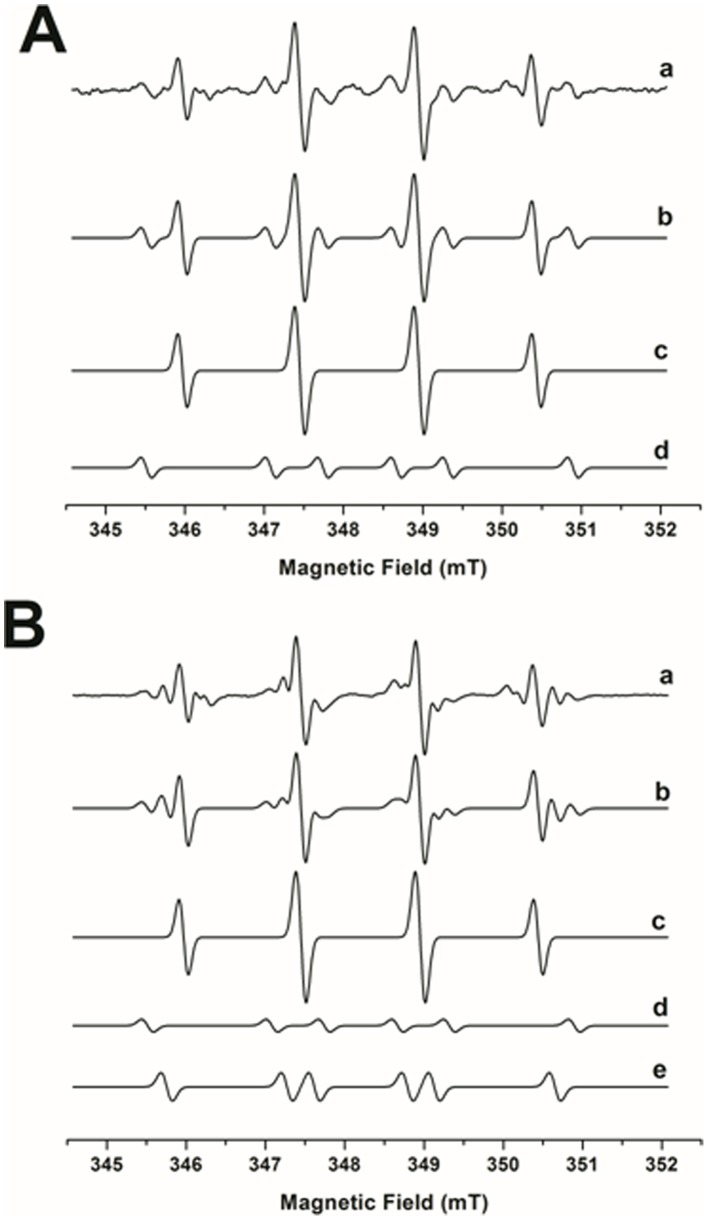
EPR spin-trapping studies and computer simulation of the AA system in the presence and absence of ferricyt *c*, under aerobic conditions. EPR spectra of DMPO-radical adducts were obtained after a 4-min incubation of (15 mM) AA at 25°C with (150 µM) cyt *c* in 50 mM phosphate buffer (pH 7.4) with (400 mM) DMPO. (A) Experimental spectrum (trace a) and computer simulations (traces b-d) of the DMPO/AA system, (B) Experimental spectrum (trace a) and computer simulations of the DMPO/AA/cyt *c* system (traces b-e). Trace *c* in panels A and B represents the DMPO-**^•^**OH adduct spectrum, and trace d can attributable to the DMPO-AA**^•^** adduct. Trace e in panel B represents an unknown DMPO adduct. Instrumental conditions: microwave power, 20.2 mW; modulation amplitude, 1.0; time constant, 1.63 s; scan rate 0.1 G/s; and receiver gain, 1.12×106.

Aiming to demonstrate generation of AA**^•^** radical by the AA/cyt *c* system, EPR spin trapping experiments with DMPO at a higher concentration (400 mM) were performed (Figure 4AB). An additional radical adduct appears in Figures 4Aa and 4Ba inside the hydroxyl radical adduct lines that may be attributable adducts derived from superoxide or peroxyl radicals [35]. On the other hand, Dutra et al. [14] previously attributed a 6-line EPR signal obtained during treatment of AA with Fe(II)EDTA in the presence of DMPO to an AA-derived secondary carbon-centered radical, probably the enoyl AA**^•^** radical generated by hydrogen abstraction from AA. Indeed, in the absence of ferricyt *c*, AA yielded two adduct signals (trace a) that were assigned by computer simulation (trace b) to DMPO-**^•^**OH **(**a_N_ = 1.51 mT; a_H_ = 1.47 mT, trace c), and DMPO-**^•^**AA (a_H_ = 2.24 mT; a_N_ = 1.59 mT, trace d) adducts (Figure 4A) [14]. In the presence of ferricyt c, an unidentified adduct with a_H_ = 1.86 mT; a_N_ = 1.53 mT (trace e) was also detected. Consistently, the α-aminoketone DAB was also found to generate the 4-line DMPO-**^•^**OH signal adduct and the putative 6-line POBN-DAB**^•^** adduct during incubation in aerated phosphate buffer [22].

### AA Promotes Direct Reduction of Ferricyt *c* Initiating the AA Aerobic Oxidation

**Figure 5:**
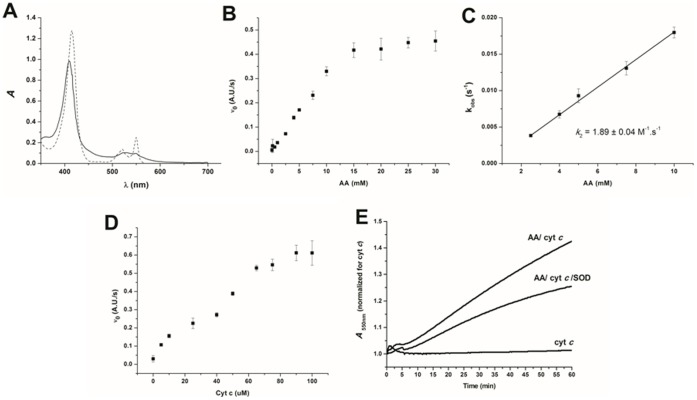
UV-Vis spectral changes in ferricyt *c* treated with AA. (A) UV-Vis spectral changes of the ferricyt *c* (10 µM)/AA (5.0 mM) system. The ferricyt *c* spectrum is shown by the solid line, and the ferricyt c/AA spectrum is shown by the dashed line. (B) Observed initial rates of the reduction of ferricyt *c* (50 µM) by AA (0.50–30 mM) monitored for 20 min. (C) Values of kobs measured at increasing concentrations of AA to calculate the k2 value. (D) The effect of cyt *c* concentration (5.0–100 µM) on the initial rate of AA (15 mM)-promoted ferricyt *c* reduction. (E) Temporal increase of the ferricyt *c* (50 µM) reduction by AA (1.0 mM) in the presence or absence of CuZnSOD (50 U/mL). All of the experiments (n = 3) were performed in Chelex-treated 50 mM phosphate buffer, pH 7.4, at 37°C.

The incubation of ferricyt *c* (10 µM) with 100- to 500-fold molar excess of AA led to UV-visible spectral changes that are characteristic of heme iron reduction to the ferrous form (Figure 5A). The initial rate of heme iron reduction by AA, monitored at 550 nm, was found to be dependent on the concentration of AA (Figure 5B), and initial rates and *k*_obs_ values were plotted as a function of AA concentration (Figure 5C), from which *k*_2_ = 1.89±0.04 M^−1^s^−1^ was calculated. This value is approximately 10-fold higher than that measured in the absence of ferricyt *c* (0.160±0.007 M^−1^s^−1^) [14]. The plot of initial rate versus ferricyt *c* concentration (Figure 5D) revealed a two-step behavior curve suggestive of two populations of cytochrome *c* reacting with AA, possibly the native form and the MG-modified cytochrome *c* form [43]. An inflexion occurred at 50 µM ferricyt *c* with the AA concentration fixed at 15 mM, which coincides with the saturation effect of AA in Figure 5B.

The initial rate of ferricyt *c* reduction by AA was only partially affected by CuZnSOD addition, which is indicative of the occurrence of the direct reduction of ferricyt *c* by the enolAA and/or AA**^•^** radical (Figure 5E). To ascertain whether AA can reduce ferricyt *c*, the reduction potential of AA was evaluated by cyclic voltammetry in DMSO containing 0.10 M tetrabutylammonium perchlorate (as a supporting electrolyte). Two reduction waves, at E_0_ (AA**^•^**/AA) = −0.51 V and E_0_ (AA**^•^**/AA_imino_) = −1.0 V (*vs.* SHE), were found (not shown). Therefore, knowing that the E_0_ of the cyt *c*.Fe^3+^/cyt *c*.Fe^2+^ pair is +0.26 against SHE [30], the reduction of ferricyt *c* by AA is, in fact, thermodynamically feasible. The measured reduction peaks of AA could conceivably correspond to the enolAA oxidation to the resonant enoylAA**^•^** radical, followed by a second electron abstraction to yield iminoAA, for which the hydrolysis culminates in the MG and NH_4_^+^ ion formation (Figure 1). On the other hand, knowing that the reduction potentials of the AA^•^/AA_imino_ (in DMSO) and H_2_O_2_/HO**^•^** (in H_2_O) pairs are −1.0 V and +0.38 V [44]), respectively, electron transfer from AA**^•^** to H_2_O_2_ leading to HO**^•^** radical generation may also be thermodynamically favorable.

Based on the data reported hereto, the mechanism of AA oxidation by ferricyt *c* (Figure 1) can be envisaged as follows:

A. In the absence of oxygen:
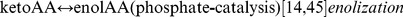
(2)

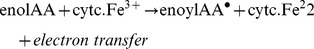
(3)

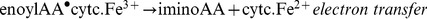
(4)

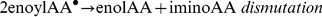
(5)

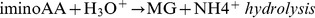
(6)


B. In the presence of oxygen, the enoyl AA radical formed by the reaction represented by Eq. 3 initiates the oxidation chain, as follows:
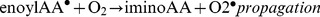
(7)

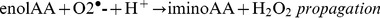
(8)

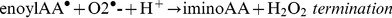
(9)

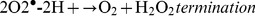
(10)

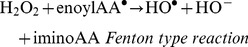
(11)


The iminoAA species (methylglyoximine) can be formed either anaerobically or in the presence of oxygen and is expected to undergo spontaneous hydrolysis to MG and NH_4_^+^ ion (Eq. 6). Ferricytochrome *c*, in turn, can also be reduced by the O_2_^•**−**^ radical. Even though it is thermodynamically favored, direct oxidation of AA (E_0_ AA/AA^•^ = −0.51 V) by molecular oxygen (O_2_^•−/^O_2_ =  −0.33 V) [46] probably does not occur because of the spin forbiddance of this process. Because the reduction potential of the enoylAA^•^/iminoAA pair was found to be −1.00 V, the enoylAA^•^ radical species can indeed reduce ferricyt *c* (Eq. 4) as well as molecular oxygen (Eq. 7), leading to iminoAA and O_2_^•**−**^, respectively. It is worth noting that the ALA-derived enoyl radical (ALA^•^), similar to the AA^•^, semiquinones, and O_2_^•**−**^ radicals, can reduce and release iron from the ferritin core, thereby amplifying potentially deleterious oxidizing free radical chains sparked by α-aminoketones [17], [47].

### Ferricyt *c* Reaction with AA Yields MG as the Final Product

The incubation of 5.0 mM AA in phosphate buffer, pH 7.4, in the absence and presence of 50 µM ferricyt *c* for 2 h in a sealed flask, under a condition of limiting oxygen concentration (approx. 200 µM), produced (39.3±1.9 *µ*M) and (45.3±4.6) MG (triplicates), respectively, after its derivatization with 1,2-diaminobenzene (See Methods). For comparison, a 2-fold higher concentration of MG (92.9±1.2 *µ*M) was detected in 30 µM Fe(II)EDTA-containing solution under the same experimental conditions. In addition, in the absence of AA, the HPLC 2-methylquinoxaline peak area was not significantly altered, attesting that the internal standard does not decompose to methylglyoxal plus 1,2-diaminobenzene under the experimental conditions. Sub-stoichiometric amounts of MG have also been reported in the SSAO-catalyzed aerobic oxidation of AA to MG [33]. Low yields of the MG product are actually expected because MG, an α-oxoaldehyde, may undergo Schiff condensation with non-reacted AA to form pyrrole derivatives. Accordingly, Soares *et al.* [22] recently reported formation of a dipyrrole adduct of DAB with its oxidation product, under experimental conditions similar to those used with AA. By the way, covalent Schiff attachment of MG to cytochrome *c*, a lysine-rich protein, has been pointed out to have biological relevance [41], [48].

### AA does not Promote Structural Alterations in Cytochrome *c* and does not Affect the Heme Iron Coordination Sphere

**Figure 6:**
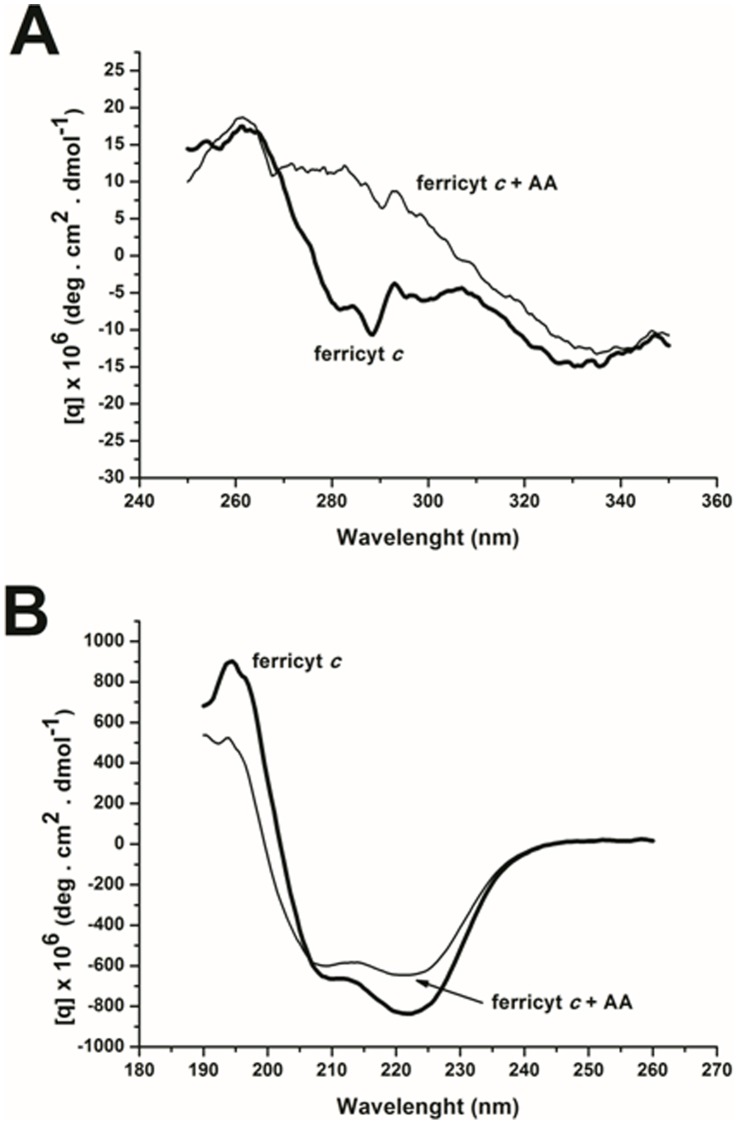
CD spectra of ferricyt *c* incubated with AA. (A) near UV, and (B) far CD spectra of (10 µM) ferricyt *c* treated with (5.0 mM) AA for 12 h. The thick black line in all of the spectra represents the ferricyt *c* control. Experimental conditions: 50 mM phosphate buffer, pH 7.4, at 37°C.

Radical intermediates, H_2_O_2_ and MG formed by AA aerobic oxidation initiated by ferricyt *c* can potentially cause protein amino acid modifications, secondary and tertiary structural changes and heme degradation [14], [49], eventually leading to partial protein denaturation and even alterations in biological functions. Electron transfer from AA to ferricyt *c* may proceed by an inner sphere mechanism involving aromatic amino acid residues of the protein [50]–[51]. However, both the Near-UV CD (Figure 6A) and the Far-UV CD spectra (Figure 6B) of ferricyt *c* in the presence of AA revealed no significant alterations in tertiary structure, the observed changes being only related to formation of ferrocyt *c*. Accordingly, the addition of ascorbate (5 µM) in the presence of ferricyt *c* resulted in similar Far and Near-UV spectra obtained for ferricyt *c* AA-treated samples. Worth to note, a significant increase (*ca.* 50%) of the fluorescence intensity (λ_em_ 340 nm, λ_exc_ 280 nm; n = 5) assignable to Trp residue oxidation was observed after 4 h of ferricyt *c* (50 µM) treatment with AA (5.0 mM) (not shown). This might indicate that electron transfer from AA to ferricyt *c* proceeds by an inner sphere mechanism involving aromatic amino acid residues of the protein [48]–[49]. Accordingly, no protein oligomerization or fragmentation was detected by 24-h SDS-PAGE experiments with AA (1.0–5.0 mM)-treated ferricyt *c* (50 µM) (data not shown).

**Figure 7:**
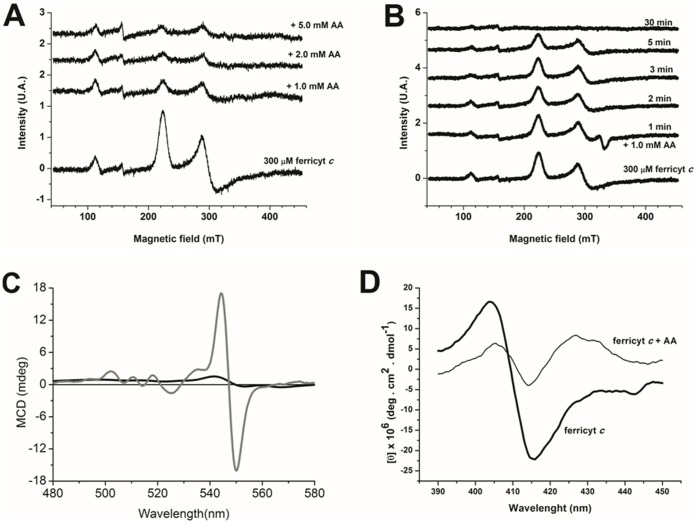
Low-temperature EPR spectra of ferricyt *c* treated with AA. (A) EPR spectra of (300 µM) ferricyt *c* after a 12-h incubation with (1.0–5.0 mM) AA. (B) Time-response ERP spectra of ferricyt *c* treated with (1.0 mM) AA for 30 min. Incubation conditions: 50 mM phosphate buffer, pH 7.4, at 37°C. (C) MCD spectra of ferricyt *c* in the presence of AA. The conditions are ferricyt *c* (40 µM) MCD spectrum before treatment with AA (thick line) and MCD ferricyt *c* spectrum after 12 h of (5.0 mM) AA treatment (thin line). (D) CD spectrum of ferricyt *c* treated with 5.0 mM AA. Incubation conditions: 50 mM phosphate buffer, pH 7.4, at 37°C.

Given that the AA/ferricyt *c* system generates free radicals when oxidized by O_2_ (Figure 3), direct EPR analysis at low temperature was also performed to investigate possible changes in the heme iron coordination sphere. The EPR spectrum of ferricyt *c* (Figure 7A) obtained before AA addition is assigned to the well-characterized low-spin form of ferricyt *c* with a rhombic structure, containing traces of oxidized prosthetic group [52]. The ferricyt *c* EPR spectrum, which was run successive times during incubation with AA, revealed only the loss of the heme iron signal, with no increase in the g = 4.3 signal (Figure 7A). The experiment depicted in Figure 7B shows that AA (1.0 mM) promotes a total reduction of the ferryl low-spin form in only 30 min of treatment. That ferricyt *c* (50 µM) is totally reduced to its ferrous form when incubated with AA (5.0 mM) at 37°C for 2 h was also confirmed by Raman spectroscopy studies, which exhibited initial and final acquired spectra that were identical to those reported for ferri- and ferrocyt *c* (not shown) [53]. Consistent with the results obtained by UV-visible spectroscopy (Figure 5B), an AA dose-dependent (1.0–5.0 mM) decrease in the iron EPR signal was observed (Figure 7B). However, the signals assigned to ferricyt *c* heme were not altered, which suggests no oxidative damage promoted by AA on the protein amino acid residues. Additional MCD and CD measurements in the wavelength region of heme absorption were also run for ferricyt *c* treated with AA and, again, resulted only in the typical ferrous heme protein signal without evidence of damage to the heme group or changes in its coordination sphere. Therefore, almost complete ferricyt *c* conversion to its ferro form by 1.0 mM AA was confirmed after 2 h of treatment (Figure 7CD), with no evidence of changes in the heme iron coordination sphere. The CD spectra of AA-treated ferricyt *c* predominantly result from the contribution of ferrous species and remnant non-reduced heme iron. With respect to the reduction mechanism of ferricyt *c* by AA, it might involve direct transfer by either an inner (coordination) or an outer (electrostatic interaction) sphere mechanism, or a transfer through the porphyrin or aromatic amino acid (Tyr, Trp) residues of the protein, similar to that long proposed by Wallace *et al.* [48] based on their study of oxyhemoglobin oxidation to methemoglobin, which was induced by several nucleophiles.

Considering that ferricyt *c* may potentially acquire peroxidase activity when exposed to AA-generated reactive radicals and MG, the effect of AA on liposome-incorporated ferricyt *c* was investigated. Incubation of native ferricyt *c* with PC/PE/CL liposomes, a mimetic of the inner mitochondrial membrane, resulted in an increase in the MDA content, which was similar to previously reported data [54]. However, MDA production in the presence of AA-treated ferricyt *c* was significantly lower (approx. 2.5 times), thus eliminating possible gains of peroxidatic activity by the cytochrome *c*. This scenario was actually predicted based on the UV-Vis, EPR, MCD, and Raman spectra, which were indicative of no changes in the cytochrome *c* coordination sphere, a response that is clearly observed when the protein displays peroxidase activity.

## Discussion

Ferricyt *c*-induced oxidation of AA by O_2_ (Figure 2) is shown here to be initiated by a one-electron transfer from AA to the heme Fe(III), yielding the resonance stabilized enoyl radical of AA, C^•^HNH_2_CO-CH_3_ ↔ CH_2_NH_2_CO**^•^**-CH_3_ (AA^•^) (Eq. 3). Subsequent electron transfer from AA**^•^** to a second ferricyt *c* molecule yields the imineAA product (CHNHCO-CH_3_) (Eq. 4), which can be hydrolyzed to form the MG and NH_4_^+^ ion (Eq. 6). This mechanism is analogous to that postulated by Castro *et al.* [55] to describe the mechanism of primary and secondary amine oxidations by ferriporphyrin complexes. These authors claimed that a -CHNH-amine moiety is essential for iron reduction. The most reactive amine studied was 2-amino-1-phenylethanone, an α-aminoketone similar to AA and ALA. No apparent rate constant of reduction of the Fe(III)porphyrin by 2-amino-1-phenylethanone in benzene was provided by the authors, who only described it as “too fast”. The extremely high reactivity of 2-amino-1-phenylethanone was attributed by the authors to its enolization and to the resulting highly stable enoyl radical, which could exhibit a higher binding constant to Fe^2+^. In this regard, we note that the H_2_PO_4_ **^−^** anion in phosphate buffer (pH 7.4) reportedly acts as a bifunctional catalyst of aldehyde and ketone enolization (Eq. 2), including isobutanal, ALA and AA, thus favoring AA oxidation [14]–[21].

Once formed, the AA**^•^** radical behaves similar to a semiquinone by undergoing dismutation (Eq. 5), transferring one electron to O_2_ yielding O_2_ **^•−^**radical (Eq. 7), and electron transfer to H_2_O_2_ rendering HO**^•^** radical (Haber-Weiss type reaction) (Eq. 11). A radical oxidation chain of AA propagated by superoxide and AA**^•^** could then take place (Eqs. 7 and 8). In contrast, in the absence of oxygen, the oxidation of AA by ferricyt *c* can still occur in two consecutive unielectronic steps, which would also generate imineAA (Eqs. 3–5), subsequently yielding MG by hydrolysis (Eq. 6), albeit at a much lower concentration than in the presence of air.

Accordingly, ferricyt *c* is shown here to increase the rate of oxygen consumption by AA (Figure 2) while being reduced to its ferro form toward oxygen depletion (Figure 4A–D). Iron reduction is also observed upon treatment of ferricyt *c* with millimolar AA under CuZnSOD addition (Figure 5E). AA could interact with the iron center simply by electrostatic attraction or by electron transfer through the porphyrin or aromatic amino acid residues of the protein, starting from affinity binding [48].

Superoxide radical and H_2_O_2_ produced during the reaction do not promote changes in the ferricyt *c* heme coordination sphere (Figure 7A–D). The reaction of cytochrome *c* with peroxides reportedly leads to the conversion of heme iron to the ferryl high-spin form with rhombic symmetry, accompanied by bleaching of the Soret band [56]. However, in the present study, the heme group was preserved from oxidative damage in spite of the generation of H_2_O_2_ and free radicals. These data could be explained by the fact that an excess of AA keeps the heme in the reduced state, which prevents the conversion of heme iron to very reactive high valence states, ultimately leading to radical generation at the heme crevice and subsequent bleaching [53]. As previously described [52], when incorporated into PC/CL vesicles, ferricyt *c* alone promotes a discrete increase (approx. two-fold) in the MDA concentration, which is indicative of polyunsaturated fatty acid peroxidation, as compared to Fe(II)EDTA (sixty-fold). The addition of AA results in a concentration-dependent inhibition of MDA formation from vesicles (not shown).

These data are consistent with the hypothesis that AA can reduce and maintain ferricyt *c* in the ferro form, hindering protein redox cycling and bleaching. Data obtained from CD, UV-Vis absorption and, Raman studies indicated that AA does not alter the cytochrome secondary and tertiary structure (Figure 6AB) and does not promote oxidative damage to the amino acid residues, although putative Trp fluorescence changes has been observed. In turn, the EPR studies conducted at a low temperature confirmed the total reduction of ferricyt *c*, with no structural changes that might impair the spectral properties of the Fe^2+^-heme (Figure 7AB). Further investigation is needed to determine whether cytochrome is modified in the course of this reaction to an as yet non-described form, with a gain or loss of a new function.

### Biological Implications

AA [14], similar to dihydroxyacetone phosphate [57], ALA [45], DAB [22] and glucosamines [58], undergoes superoxide-propagated oxidation to an α-oxoaldehyde and H_2_O_2_, both of which are known to be reactive species implicated in normal and adverse biological events. Methylglyoxal, the oxidation product of AA, was found to be approximately 7 fold and 6 fold higher in the plasma of type 1 and type 2 diabetes patients, respectively, than in normal individuals (96.3±9.5 nM) [59] and was identified as an apoptotic inductor [60]. Although detected in biological samples at sub-micromolar concentrations, MG is currently recognized as a strong electrophile that can form advanced glycation end products (AGEs) when reacting with various proteins and enzymes, and ethane adducts with nucleobases [11]. Chemical modifications of glycolytic enzymes such as aldolase and glyceraldehyde-3-phosphate dehydrogenase [61] have been credited to MG. It has also been found to depress reduced glutathione in cultures of rat hepatocytes [62], possibly via inhibition of glutathione reductase [63] and glutathione peroxidase [64], and to induce cellular swelling and apoptosis of pancreatic β-cells [65], [66]. Furthermore, MG reportedly produces free radicals during the glycation of amino acids, including alanine [67], and superoxide radicals from its reaction with guanidine compounds [68].

On the other hand, H_2_O_2_ was related to the development of diabetes [69] and to the impairment of glucose-stimulated insulin secretion by pancreatic islets in female albino rats [70]. In addition, elevated iron and copper ion concentrations were reported to occur in the plasma of type II diabetics [1], probably released by metal storage proteins such as ferritin and ceruloplasmin, respectively.

Considering that (i) threonine and glycine catabolism have been evoked as a source of AA [71] and ∼10% of methylglyoxal production [29], [72], (ii) rat liver threonine dehydrogenase appears to be involved in 87% of the degradation of hepatic threonine [29], (iii) mitochondrial threonine dehydrogenase appears to be linked to aminoacetone synthetase [73], (iv) the enol form predominates over the keto form in tautomeric equilibrium of carbonyl compounds in aprotic media [21], and (v) AA is reportedly synthesized in the mitochondrial matrix [14], it is conceivable that enolAA diffuses across the lipid phase of the inner mitochondrial membrane and reaches cytochrome *c* at the outer side, leading to the hemeprotein oxidative injury.

## Conclusions

AA, a putative threonine and glycine catabolite reportedly overproduced in diabetes, is shown here to reduce ferricyt *c* directly to the ferro form *in vitro* in both anaerobic and aerobic conditions. Two one-electron transfer steps from AA to ferricyt *c* afford methylglyoxal imine (iminoAA), for which hydrolysis yields MG and NH_4_ **^+^** ions. In the presence of molecular oxygen, the conversion of AA to MG is propagated by O_2_ **^•−^** and enoylAA**^•^** radicals, leading to the formation of H_2_O_2_ as well. Although they are known as highly reactive catabolites, MG and H_2_O_2_ do not cause cytochrome *c* heme destruction and do not significantly affect the protein’s secondary and tertiary structure. If it were to occur in diabetics, the direct reduction of cytochrome *c* by excess AA might contribute to the impairment of mitochondrial functions by inhibiting key enzymes, interfering in ATP synthesis, and participating in apoptotic death.
